# Supplementary material for: Hybrid Models and Biological Model Reduction with PyDSTool
Source: PLoS Comput Biol. 2012 Aug 9;8(8):e1002628. doi: 10.1371/journal.pcbi.1002628 (PMC3415397; doi:10.1371/journal.pcbi.1002628)
Supplement: Text S4 — Complete source code for the PyDSTool package (version 0.88.120504). Includes API documentation and help files linking to web pages. This file is identical to the current public release on Sourceforge.net. (ZIP) [file pcbi.1002628.s004.zip › PyDSTool/html/toc-PyDSTool.Toolbox.event_driven_simulator-module.html]

xml version="1.0" encoding="ascii"?


event\_driven\_simulator


# Module event\_driven\_simulator

---

## Classes

FIFOqueue\_uniquenode

composed\_map1D  
 connection  
 delay\_map  
 identity\_map  
 map  
 map1D  
 map2D  
 node  
 simulator  

## Functions

extract\_digraph  

head

sequences\_to\_eventlist

tail

## Variables

API

API\_class  

Continuous

Discrete

DomainType  

Inf

LargestInt32

Macheps  

NaN

\_all\_complex

\_all\_float

\_all\_int

\_all\_numpy\_complex

\_all\_numpy\_float

\_all\_numpy\_int

\_classes

\_complex\_types

\_float\_types

\_functions

\_instances

\_int\_types

\_num\_equivtype

\_num\_maxmin

\_num\_name2equivtypes

\_num\_name2type

\_num\_type2name

\_num\_types

\_pytypefromtype

\_real\_types

\_seq\_types

all  
 alltrue  
 any  
 argmax  
 argmin  
 atleast\_1d  
 clip  
 complex  
 complex128  
 complex192  
 complex64  
 complex\_  
 complexfloating  
 deepcopy  
 double  
 exp  
 finfo  
 fit\_function  
 float  
 float32  
 float64  
 float96  
 float\_  
 floating  
 greater  
 idmap  
 int  
 int0  
 int16  
 int32  
 int64  
 int8  
 int\_  
 integer  
 intersect  

isfinite

less  
 less\_equal  
 log  
 logical\_or  
 mat  
 minpack  
 ndarray  
 norm  
 npy  

null\_predicate

null\_predicate\_class  
 ones  
 os  
 predicate\_op  
 putmask  
 searchsorted  
 shape  
 sign  
 sometrue  
 spy  
 sqrt  
 swapaxes  
 sys  
 take  

targetLangs

time  
 types  
 zeros  

---

[hide private]
